# Patient Age and Survival After Surgery for Esophageal Cancer

**DOI:** 10.1245/s10434-020-08653-w

**Published:** 2020-05-28

**Authors:** Jesper Lagergren, Matteo Bottai, Giola Santoni

**Affiliations:** 1 Upper Gastrointestinal Surgery, Department of Molecular Medicine and Surgery, Karolinska Institutet, Karolinska University Hospital, Stockholm, Sweden; 2 School of Cancer and Pharmaceutical Sciences, King’s College London, London, UK; 3 Division of Biostatistics, Institute of Environmental Medicine, Karolinska Institutet, Stockholm, Sweden

## Abstract

**Background:**

Esophagectomy for esophageal cancer is associated with a substantial risk of life-threatening complications and a limited long-term survival. This study aimed to clarify the controversial questions of how age influences short-term and long-term survival.

**Methods:**

This population-based cohort study included almost all patients who underwent curatively intended esophagectomy for esophageal cancer in Sweden in 1987–2010, with follow-up through 2016. The exposure was age, analyzed both as a continuous and categorical variable. The probability of mortality was computed using a novel flexible parametric model approach. The reported probabilities are proper measures of the risk of dying, and the related odds ratios (OR) are therefore more suitable measures of association than hazard ratios. The outcomes were 90-day all-cause mortality, 5-year all-cause mortality, and 5-year disease-specific mortality. A novel flexible parametric model was used to derive the instantaneous probability of dying and the related OR along with 95% confidence intervals (CIs), adjusted for sex, education, comorbidity, tumor histology, pathological tumor stage, and resection margin status.

**Results:**

Among 1737 included patients, the median age was 65.6 years. When analyzed as a continuous variable, older age was associated with slightly higher odds of 90-day all-cause mortality (OR 1.05, 95% CI 1.02–1.07), 5-year all-cause mortality (OR 1.02, 95% CI 1.01–1.03), and 5-year disease-specific mortality (OR 1.01, 95% CI 1.01–1.02). Compared with patients aged < 70 years, those aged 70–74 years had no increased risk of any mortality outcome, while patients aged ≥ 75 years had higher odds of 90-day mortality (OR 2.85, 95% CI 1.68–4.84), 5-year all-cause mortality (OR 1.56, 95% CI 1.27–1.92), and 5-year disease-specific mortality (OR 1.38, 95% CI 1.09–1.76).

**Conclusions:**

Patient age 75 years or older at esophagectomy for esophageal cancer appears to be an independent risk factor for higher short-term mortality and lower long-term survival.

**Electronic supplementary material:**

The online version of this article (10.1245/s10434-020-08653-w) contains supplementary material, which is available to authorized users.

The most effective treatment for locally advanced esophageal cancer is surgical resection (esophagectomy) combined with chemo(radio)therapy.^1^ Esophagectomy is an extensive procedure that carries a high risk (40–60%) of serious and sometimes lethal complications.^1^ The long-term (5-year) survival after surgery is limited—only 31% according to nationwide population-based studies from Sweden.^2^,^3^ The short-term safety and long-term benefits of esophagectomy in older people is controversial. Some studies have found no clear differences in short- or long-term mortality after esophagectomy when comparing patients older or younger than 70 years of age,^4^–^10^ but most of these studies were based on few and possibly selected patients from single centers. On the other hand, a number of other studies as well as a meta-analysis found an increased risk of short- and long-term mortality in elderly patients, although some of these studies also included patients who did not undergo curatively intended oesophagectomy.^11^–^21^ Thus, the role of age in relation to mortality after esophagectomy for esophageal cancer remains uncertain. If patient age independent of other factors influenced the survival after surgery it should be taken into account in the clinical decision making, i.e. when deciding whether esophagectomy should be recommended or not.

We hypothesized that the short-term mortality was higher in older patients than in younger patients, while the long-term survival was independent of age. With the aim of testing these hypotheses, the present study took advantage of a well-established nationwide Swedish cohort with comprehensive data on key variables and complete follow-up for mortality. We availed ourselves of a novel statistical method that allowed estimation of mortality risk, properly defined as the probability of dying at any given time point.

## Methods

### Design

We used data from a Swedish, nationwide, population-based cohort study that has been previously validated and described in detail elsewhere.^22^,^23^ Briefly, this study included all patients with esophageal cancer treated with curative intent between 1987 and 2010, with follow-up until November 2014. From the Swedish Cancer Registry, patients with a diagnosis of esophageal cancer (150.0, 150.8, or 150.9) between 1987 and 2010 were identified according to the 7th edition of the International Classification of Diseases (ICD7). Patients with esophageal cancer who underwent esophagectomy were identified from the Swedish Patient Registry. The Swedish Causes of Death Registry was used to provide accurate data about the dates and causes of death. If the diagnosis of esophageal cancer was listed as a cause of death, then mortality was defined as disease-specific. The final source cohort consisted of 1820 patients who underwent curatively intended esophagectomy for esophageal adenocarcinoma or squamous cell carcinoma in Sweden between 1987 and 2010. They were followed up through 2016, thus allowing 5-year follow-up of all participants. After exclusion of patients with missing information on education (*n* = 69, 3.8%) or tumor stage (*n* = 14, 0.8%), the final sample included 1737 (95.4%) patients. The data were retrieved from medical records and five national Swedish health and population registries: Cancer Registry, Patient Registry, Cause of Death Registry, Prescribed Drug Registry, and Longitudinal Integration Database for Health Insurance and Labor Market Studies (LISA). The linkages of individuals’ data were enabled by the personal identity numbers given to each Swedish resident upon birth or immigration.^24^ The study was approved by the Regional Ethical Review Board in Stockholm, Sweden.

### Exposure

The study exposure was the patient’s age at the date of surgery. This information was available in all registries and the medical records.

### Outcomes

The main outcome was 5-year all-cause mortality, which was selected because the 5-year cut-off reduces the influence of death due to factors unrelated to the esophageal cancer or its treatment, and the all-cause survival is the most complete and accurate assessment of survival. Secondary outcomes were 90-day all-cause mortality and disease-specific 5-year mortality. The choice of 90 days as the short-term mortality cut-off was based on research showing a time shift of postoperative mortality from 30 days to 31–90 days with more recent calendar time.^25^ The definition of disease-specific mortality was mortality with an esophageal cancer diagnosis as a primary or contributing cause of death after excluding the first 90 days of surgery. The data on all mortality outcomes were retrieved from the Cause of Death Registry, which has over 99% completeness for date and cause of death.^26^

### Covariates

The confounders were seven variables known to be associated with both the exposure (age) and the outcome (mortality): (1) sex (male or female); (2) attained education level (≤ 9 years, 10–12 years, or > 12 years of formal education); (3) comorbidity (Charlson comorbidity index score 0, 1, or ≥ 2);^27^ (4) neoadjuvant therapy (typically chemoradiotherapy, no or yes); (5) tumor histology (adenocarcinoma or squamous cell carcinoma); (6) pathological tumor stage (0–II or III–IV); and (7) resection margin status (no tumor involved [R0] or tumor involved [R1 or R2]). Data on sex were retrieved from the medical records and all the registries used. Information on comorbidities was collected from the Patient Registry, which has a high completeness and accuracy of recording of diagnoses,^28^ while information on neoadjuvant therapy, tumor histology, pathological tumor stage, and resection margin status was obtained from review of all relevant surgery charts and histopathology records from the Swedish hospitals where the esophagectomy had been conducted.^22^,^23^

### Statistical Analysis

The probability of mortality was computed using a novel flexible parametric model approach^29^,^30^ and the results were displayed graphically. The probability of death was defined as the average probability of dying in an infinitely short time interval. This quantity is related to the hazard function through the following mathematical expression: *g*(*t*) = 1 − exp[–*h*(*t*)], where *g*(*t*) is the probability function, *h*(*t*) is the hazard function, and exp is the exponential function (see working paper at http://www.imm.ki.se/biostatistics/eventprob/Working_paper_2020.pdf). This quantity can be readily estimated using general parametric survival models with a user-defined baseline hazard function. Odds ratios (OR) of death as a function of both a linear age variable and a categorical age variable were reported. For comparison, flexible semi-parametric models were used to compute hazard ratios (HR) as a function of age at surgery. All analyses were conducted using the user-written Stata program *stpreg* available at http://www.imm.ki.se/biostatistics/eventprob.

We first analyzed age as a continuous variable in relation to the mortality outcomes. Several models with different age and time functions, and age and time interactions, were tested and the final model was selected based on the Akaike’s information criterion. Age was introduced into the model as a linear predictor and through restricted cubic splines. Time since surgery was modeled with restricted cubic splines. The interaction between the exposure and time was also tested. Second, we analyzed age in three categories (< 70, 70–74, and ≥ 75 years) in relation to the probability of dying. The cut-off of 70 years of age was used because it is the most common cut-off used in the literature. The cut-off of 75 years of age was introduced to examine associations among older people.

Patients entered the study at the date of surgery and remained in the cohort until the date of death or end of the study (31 December 2016), whichever occurred first. For each of the three mortality outcomes, we analyzed a crude model and an adjusted model. The adjusted model included the seven confounders presented above, using the same categorization. Stratified analyses were carried out for sex, tumor histology, pathological tumor stage, and resection margin status.

## Results

### Patients

Characteristics of the 1737 patients included in the study are described in Table 1. Median age at surgery was 65.6 years (interquartile range 5.6–71.9 years), and the age range was 18–89 years.

**Table 1:** Sociodemographic and clinical characteristics of participants who have undergone esophagectomy for esophageal cancer in Sweden

Characteristic	*N* (%)
Total	1737 (100.0)
*Age, years*	
<60	505 (29.1)
60–69	655 (37.7)
70–74	340 (19.6)
75+	237 (13.6)
*Sex*	
Males	1306 (75.2)
Females	431 (24.8)
*Formal education, years*	
≤9	889 (51.2)
10–12	624 (35.9)
>12	224 (12.9)
*Charlson comorbidity index*	
0	964 (55.5)
1	491 (28.3)
2+	282 (16.2)
*Neoadjuvant therapy*	
No	1175 (67.6)
Yes	562 (32.3)
*Tumor histology*	
Adenocarcinoma	769 (44.3)
Squamous cell carcinoma	968 (55.7)
*Pathological tumor stage*	
0–II	1042 (60.0)
III–IV	695 (40.0)
*Resection margin status*	
R0	1470 (84.6)
R1-R2	267 (15.4)
*Deaths*	
90-day all-cause	195 (11.1)
5-year all-cause	1299 (74.8)
5-year disease-specific	1004 (65.1)

### 90-Day All-Cause Mortality

**Fig. 1:**
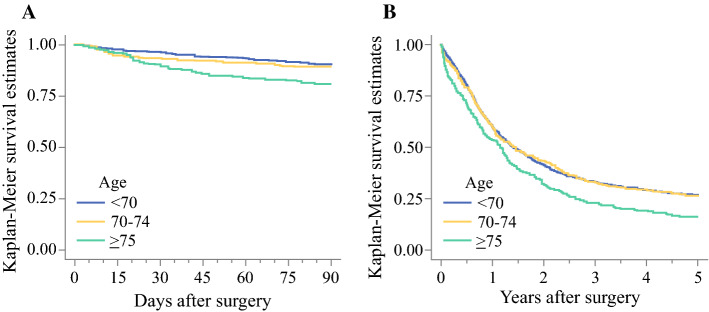
Survival estimates of age in relation to **a** 90-day all-cause mortality; and **b** 5-year all-cause mortality after esophagectomy for esophageal cancer

**Fig. 2:**
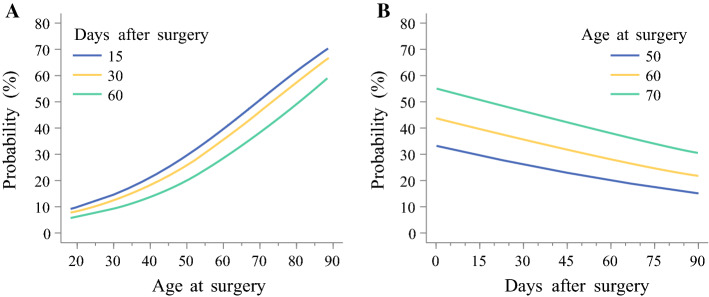
Probability of 90-day all-cause mortality after esophagectomy for esophageal cancer. **a** Probability versus age at surgery for three time points. **b** Probability versus years from surgery for three age groups

The overall 90-day mortality rate was 11.2% (*n* = 195), and 90% of patients aged below 70 years and 85% of those aged 75 years or older were alive 90 days after surgery (Fig. 1a). Figure 2 shows the changes in probability of dying within 90 days of surgery, depending on age or days after surgery. The probability of 90-day mortality increased from 10% at age 60 years to 60% or above at age 89 years (Fig. 2a). The odds of dying increased by 5% with each year of age (OR 1.05, 95% confidence interval [CI] 1.02–1.07) (Table 2). Patients aged 75 years or older had almost three times the odds of dying compared with patients younger than 70 years of age (OR 2.85, 95% CI 1.68–4.31). There was no statistically significant interaction between age and any of the seven covariates included in the model.

**Table 2:** Odds ratios, with 95% confidence intervals, of age in relation to mortality outcomes after esophagectomy for esophageal cancer

	Deaths [*N* (%)]	Unadjusted OR (95% CI)	Adjusted OR (95% CI)
*90*-*day all*-*cause mortality*^*a*^			
Age, linear	195 (11)	1.05 (1.02–1.07)	1.05 (1.02–1.07)^b^
Age, years			
< 70	113 (10)	1.00 (reference)	1.00 (reference)^b^
70–74	37 (11)	1.15 (0.72–1.83)	1.19 (0.72–1.96)^b^
≥ 75	45 (19)	2.63 (1.60–4.31)	2.85 (1.68–4.84)^b^
*5*-*year all*-*cause mortality*^*c*^			
Age, linear	1299 (75)	1.02 (1.01-1.03)	1.02 (1.01–1.03)^b^
Age, years			
< 70	849 (73)	1.00 (reference)	1.00 (reference)^b^
70–74	251 (74)	1.02 (0.86–1.21)	1.05 (0.88–1.26)^b^
≥ 75	199 (84)	1.49 (1.22–1.81)	1.56 (1.27–1.92)^b^
*5*-*year disease*-*specific mortality*^*d*^			
Age, linear	1004 (65)	1.01 (1.00–1.02)	1.01 (1.01–1.02)^e^
Age, years			
< 70	673 (64)	1.00 (reference)	1.00 (reference)^e^
70–74	193 (64)	0.98 (0.81–1.19)	1.06 (0.87–1.30)^e^
≥ 75	138 (72)	1.28 (1.02–1.61)	1.38 (1.09–1.76)^e^

*OR* odds ratio, *CI* confidence interval

^a^Linear function of survival time

^b^Adjusted for sex, education, Charlson comorbidity index, tumor histology, pathological tumor stage, and resection margin status

^c^Spline function of time after surgery with 3 knots

^d^Spline function of time after surgery with 4 knots

^e^Adjusted for sex, education, neoadjuvant therapy, tumor histology, pathological tumor stage, and resection margin status

### 5-Year All-Cause Mortality

**Fig. 3:**
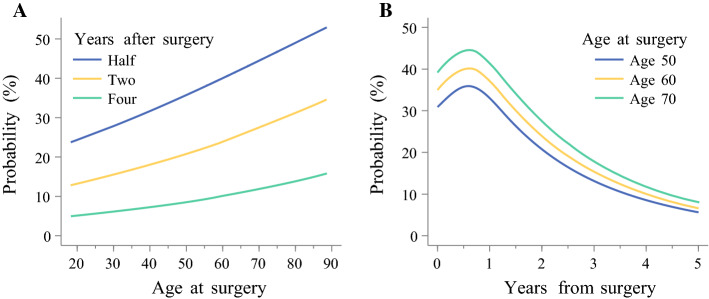
Probability of 5-year all-cause mortality after esophagectomy for esophageal cancer. **a** Probability versus age at surgery for three time points after esophagectomy for esophageal cancer. **b** Probability versus years from surgery for three age groups

The 5-year all-cause mortality rate was 74.8% (*n* = 1299). At 5 years after surgery, 27% of patients aged below 70 years and 16% of those aged 75 years or older were alive (Fig. 1b). The association between age and probability of dying is depicted in Fig. 3. The probability of death increased with age (Fig. 3a), but the strength of the association decreased with time after surgery (Fig. 3b). The adjusted odds of 5-year mortality increased by 2% with each year of age (OR 1.02, 95% CI 1.01–1.03) (Table 2). Patients aged 75 years or older had 56% higher odds of dying than those younger than 70 years of age (OR 1.56, 95% CI 1.27–1.92) (Table 2). No interaction term between age and sex, tumor histology, tumor stage, or resection margin status was statistically significant. In the stratified analyses, the strength of the association between age and 5-year all-cause mortality was stronger in patients with clear resection margins (R0) (Table 3). The results remained similar after excluding the first 90 days after surgery (electronic supplementary Table 4 and electronic supplementary Table 5).

**Table 3:** Odds ratios, with 95% confidence intervals, of age in relation to mortality after esophagectomy for esophageal cancer, stratified by sex or tumor characteristics

	90-day mortality		5-year all-cause mortality	
	Deaths [*N* (%)]	OR (95% CI)^a^	Deaths [*N* (%)]	OR (95% CI)^a^
*Sex*				
Males	155 (12)		991 (76)	
Age < 70 years	91 (10)	1.00 (reference)	661 (74)	1.00 (reference)
Age 70–74 years	30 (12)	1.29 (0.73–2.28)	191 (77)	1.11 (0.90–1.36)
Age ≥ 75 years	34 (20)	2.71 (1.47–4.99)	139 (82)	1.40 (1.10–1.79)
Females	40 (9)		431 (71)	
Age < 70 years	22 (8)	1.00 (reference)	188 (69)	1.00 (reference)
Age 70–74 years	7 (8)	0.90 (0.31–2.62)	60 (66)	0.92 (0.65–1.31)
Age ≥ 75 years	11 (16)	3.26 (1.17–9.09)	60 (88)	2.01 (1.37–2.94)
*Tumor histology*				
Adenocarcinoma	69 (9)		523 (68)	
Age < 70 years	35 (7)	1.00 (reference)	312 (65)	1.00 (reference)
Age 70–74 years	16 (9)	1.31 (0.62–2.79)	121 (71)	1.19 (0.92–1.55)
Age ≥ 75 years	18 (15)	2.79 (1.27–6.11)	90 (76)	1.44 (1.08–1.94)
Squamous cell carcinoma	126 (13)		776 (80)	
Age < 70 years	78 (11)	1.00 (reference)	537 (79)	1.00 (reference)
Age 70–74 years	21 (12)	1.09 (0.56–2.14)	130 (77)	0.93 (0.73–1.20)
Age ≥ 75 years	27 (23)	2.93 (1.43–6.00)	109 (92)	1.70 (1.27–2.28)
*Pathological tumor stage*				
0–II	108 (10)		671 (64)	
Age < 70 years	65 (9)	1.00 (reference)	439 (63)	1.00 (reference)
Age 70–74 years	19 (10)	0.98 (0.50–1.92)	122 (51)	0.96 (0.76–1.21)
Age ≥ 75 years	24 (17)	2.24 (1.13–4.45)	110 (78)	1.65 (1.28–2.14)
III–IV	87 (12)		628 (90)	
Age < 70 years	48 (10)	1.00 (reference)	410 (90)	1.00 (reference)
Age 70–74 years	18 (13)	1.53 (0.71–3.29)	129 (91)	1.21 (0.91–1.62)
Age ≥ 75 years	21 (22)	4.04 (1.76–9.24)	89 (93)	1.40 (1.00–1.96)
*Resection margin status*				
R0	135 (9)		1044 (71)	
Age < 70 years	73 (8)	1.00 (reference)	664 (69)	1.00 (reference)
Age 70–74 years	29 (10)	1.27 (0.74–2.20)	211 (71)	1.02 (0.84–1.23)
Age ≥ 75 years	33 (16)	2.75 (1.55–4.90)	169 (82)	1.61 (1.29–2.00)
R1–R2	60 (22)		255 (95)	
Age < 70 years	40 (21)	1.00 (reference)	185 (95)	1.00 (reference)
Age 70–74 years	8 (20)	0.80 (0.24–2.67)	40 (98)	1.51 (0.83–2.77)
Age ≥ 75 years	12 (37)	3.68 (0.91–14.99)	30 (94)	1.18 (0.64–2.16)

*OR* odds ratio, *CI* confidence interval

^a^ Adjusted for sex, education, Charlson comorbidity index, tumor histology, pathological tumor stage, and resection margin status

### 5-Year Disease-Specific Mortality

The 5-year disease-specific mortality rate was 76.7% (*n* = 1183). The adjusted odds of disease-specific death increased by 1% for each year of age (OR 1.01, 95% CI 1.01–1.02) (Table 2). The association between age and probability of dying showed a pattern similar to that of the 5-year all-cause mortality (electronic supplementary Fig. 4). There was no difference between the 70–74 years age group and younger patients, while patients aged 75 years or older had 38% higher odds of dying than patients younger than 70 years of age (adjusted OR 1.38, 95% CI 1.09–1.76). There was no statistically significant interaction between age and any of the covariates in the model. In the stratified analyses, older age was associated with higher odds of 5-year disease-specific mortality in participants with clear resection margins (R0) and in patients with early-stage tumor (pathological tumor stage 0, I, or II) [electronic supplementary Table 6].

### Comparison Between Odds Ratios and Hazard Ratios

Electronic supplementary Table 7 reports the powers of the probability of all-cause 90-day and 5-year mortality, which were equal to the ratios of the hazard of dying. Powers and ORs of dying were similar for values of the odds and of the hazards closer to one.

## Discussion

This study indicated that both short- and long-term mortality increased with older age of patients after esophagectomy for esophageal cancer, independent of other prognostic factors, including comorbidity and tumor stage. The probability of mortality was higher for patients aged 75 years or older at the time of surgery, particularly for short-term mortality. The association between older age at surgery and higher probability of dying was similar in the different strata of the cohort, but the 5-year mortality outcomes were more pronounced in patients with clear resection margins (R0) compared with those with tumor-involved margins (R1 or R2).

Among the methodological strengths of this study is the population-based design with inclusion of almost all patients in Sweden who underwent curatively intended esophagectomy for cancer. Data on the exposure (patient age) and outcomes (mortality) were complete and valid. The extensive clinical information from all hospitals in Sweden and nationwide registries allowed for adjustment for the main confounding factors and counteracted loss to follow-up. However, the study had no direct information about other potential confounders, such as smoking and obesity, which increase with age.^31^,^32^ Yet, these factors were partially controlled for by adjusting for comorbidities associated with smoking and obesity. A possible stricter selection of older patients considered fit enough to undergo esophagectomy or with tumors of less advanced stage might have introduced bias. However, current protocols for curative esophagectomy state that the selection for surgery should be independent of age, and the adjustment for comorbidity and tumor stage should have counteracted any such bias. The postoperative survival rates have improved during the long study period, but it is less likely that the study period influenced the association between age and survival.

The main finding of this study was that older age increases the probability of dying not only in the short-term but also in the longer term. Only some of the association between older age and 5-year mortality was explained by the higher 90-day mortality. These results are in line with other studies that found increased in-hospital mortality and 5-year mortality in patients aged 70 years or older.^15^–^17^,^19^ Older age was also associated with an increased disease-specific mortality in the present study. A previous study suggested that older age does not increase the risk of 5-year disease-specific mortality, but increases the non-surgical complication rate and susceptibility to in-hospital mortality compared with younger counterparts.^11^ Other studies have found no influence of age on long- or short-term mortality.^4^–^10^ A systematic review and meta-analysis found that age had little influence on mortality after oesophagectomy;^33^ however, some of the studies included in the meta-analysis contained few participants.^5^,^7^,^9^

The association between age and mortality in this study was not strongly affected by other factors such as comorbidity or tumor characteristics. For the 5-year all-cause mortality, the association was stronger in patients with early-stage tumors and those who had surgery with tumor-free resection margins (R0), i.e. in those with more readily curable tumors. The lack of influence of age in patients with advanced tumor stage and non-radical resection could be explained by the fact that the vast majority of these patients died from tumor recurrence independent of age.

These findings may influence clinical decision making. The study suggests that age is an independent risk factor for worse survival, both in the short- and long-term, after esophageal cancer surgery. Thus, when patients are considered for surgery or other treatment options, e.g. definite chemo(radio)therapy or a palliative route, age should be regarded as an independent prognostic factor in the clinical setting. This has not been made clear in the existing literature.

The statistical method used in this paper allowed for obtaining curves for the probability of dying throughout the follow-up time. This method can be applied to any study where the aim is to analyze the time to an event of interest, such as diagnosis of cancer and onset of a disease. Traditionally, in these types of studies, the analysis focuses on hazard ratios and hazard functions. The reported probabilities, unlike hazards, are proper measures of the risk of dying, and the related ORs are therefore more suitable measures of association than HRs. The reported probabilities are not average probabilities of dying within a given interval of time, but rather the instantaneous probabilities of dying at any given time. Because obtaining instantaneous probabilities and their ORs is simple to interpret, we recommend calculating and reporting these instead of the traditional hazards.^34^

## Conclusion

This nationwide and population-based cohort study indicates that patient age 75 years or older should be taken into consideration as an independent poor prognostic factor when patients are considered for esophagectomy for esophageal cancer. Surgery also remains the best available treatment for older patients, but age should be part of the informed consent process.

## Electronic Supplementary Material

Below is the link to the electronic supplementary material.

Supplementary material 1 (DOCX 139 kb)
