# Supplementary material for: Patient Age and Survival After Surgery for Esophageal Cancer
Source: Ann Surg Oncol. 2020 May 28;28(1):159–66. doi: 10.1245/s10434-020-08653-w (PMC7752878; doi:10.1245/s10434-020-08653-w)
Supplement: Supplementary file 1 — Supplementary material 1 (DOCX 139 kb) [file 10434_2020_8653_MOESM1_ESM.docx]

# Supplementary material

**Table 4. Odds ratios (OR) and 95% confidence intervals (CI) of age in relation to 5-year all-cause mortality after esophagectomy for esophageal cancer. Entry time 90-days after surgery.**

|  | **Deaths**  **Number (%)** | **Unadjusted**  **OR (95% CI)** | **Adjusted^*^**  **OR (95% CI)** |
| --- | --- | --- | --- |
| **Age (linear)** | 1104 (72%) | 1.01 (1.01-1.02) | 1.02 (1.01-1.02) |
| **Age (categorical)** |  |  |  |
| **<70** | 736 (70%) | 1.00 (reference) | 1.00 (reference) |
| **70-74** | 214 (71%) | 1.00 (0.83-1.20) | 1.05 (0.87-1.28) |
| **≥75** | 154 (80%) | 1.33 (1.07-1.65) | 1.40 (1.11-1.76) |

^*^Adjusted for sex, education, tumor histology, pathological tumor stage, and resection margin status.

Spline function of survival time with 4 knots.

**Table 5. Odds ratios (OR) and 95% confidence intervals (CI) of age in relation to 5-year all-cause mortality after esophagectomy for esophageal cancer stratified by sex or tumor characteristics. Entry time 90-days after surgery.**

|  | **Deaths**  **Number (%)** | **OR (95% CI)*** |
| --- | --- | --- |
| **Sex** |  |  |
| **Men (n=1151)** | 836 (73%) |  |
| Age <70 | 570 (72%) | 1.00 (reference) |
| Age 70-74 | 161 (74%) | 1.10 (0.88-1.37) |
| Age ≥75 | 105 (78%) | 1.24 (0.94-1.63) |
| **Women (n=391)** | 268 (69%) |  |
| Age <70 | 166 (66%) | 1.00 (reference) |
| Age 70-74 | 53 (63%) | 0.95 (0.66-1.39) |
| Age ≥75 | 49 (86%) | 1.87 (1.23-2.84) |
| **Tumor histology** |  |  |
| **Adenocarcinoma (n=700)** | 454 (65%) |  |
| Age <70 | 277 (62%) | 1.00 (reference) |
| Age 70-74 | 105 (68%) | 1.21 (0.92-1.59) |
| Age ≥75 | 72 (72%) | 1.32 (0.96-1.83) |
| **Squamous cell carcinoma (n=842)** | 650 (77%) |  |
| Age <70 | 459 (76%) | 1.00 (reference) |
| Age 70-74 | 109 (74%) | 0.92 (0.70-1.20) |
| Age ≥75 | 82 (89%) | 1.50 (1.08-2.08) |
| **Pathological tumor stage** |  |  |
| **0-II (n=934)** | 563 (60%) |  |
| Age <70 | 374 (59%) | 1.00 (reference) |
| Age 70-74 | 103 (57%) | 0.96 (0.75-1.24) |
| Age ≥75 | 86 (74%) | 1.57 (1.18-2.08) |
| **III-IV (n=608)** | 541 (89%) |  |
| Age <70 | 362 (88%) | 1.00 (reference) |
| Age 70-74 | 111 (90%) | 1.20 (0.88-1.63) |
| Age ≥75 | 68 (91%) | 1.15 (0.79-1.67) |
| **Resection margin status** |  |  |
| **R0 (n=1335)** | 909 (68%) |  |
| Age <70 | 591 (66%) | 1.00 (reference) |
| Age 70-74 | 182 (67%) | 1.01 (0.82-1.23) |
| Age ≥75 | 136 (79%) | 1.48 (1.17-1.89) |
| **R1-R2 (n=207)** | 195 (94%) |  |
| Age <70 | 145 (94%) | 1.00 (reference) |
| Age 70-74 | 22 (97%) | 1.87 (0.92-3.80) |
| Age ≥75 | 18 (90%) | 0.80 (0.39-1.63) |

* Adjusted for sex, education, tumor histology, pathological tumor stage, and resection margin status. Spline function of survival time with 4 knots.**Table 6. Odds ratios (OR) and 95% confidence intervals (CI) of age in relation to 5-year disease-specific mortality after esophagectomy for esophageal cancer stratified by sex or tumor characteristics.**

|  | **Deaths**  **Number (%)** | **OR (95% CI)*** |
| --- | --- | --- |
| **Sex** |  |  |
| **Men (n=1151)** | 759 (70%) |  |
| Age <70 | 515 (65%) | 1.00 (reference) |
| Age 70-74 | 147 (67%) | 1.14 (0.90-1.45) |
| Age ≥75 | 94 (70%) | 1.24 (0.93-1.66) |
| **Women (n=391)** | 248 (63%) |  |
| Age <70 | 158 (63%) | 1.00 (reference) |
| Age 70-74 | 46 (55%) | 0.87 (0.59-1.29) |
| Age ≥75 | 44 (77%) | 1.76 (1.14-2.72) |
| **Tumor histology** |  |  |
| **Adenocarcinoma (n=700)** | 403 (58%) |  |
| Age <70 | 247 (56%) | 1.00 (reference) |
| Age 70-74 | 96 (62%) | 1.29 (0.97-1.73) |
| Age ≥75 | 60 (60%) | 1.23 (0.87-1.75) |
| **Squamous cell carcinoma (n=842)** |  |  |
| Age <70 | 601 (71%) | 1.00 (reference) |
| Age 70-74 | 426 (71%) | 0.88 (0.66-1.17) |
| Age ≥75 | 97 (66%) | 1.57 (1.13-2.20) |
| **Pathological tumor stage** |  |  |
| **0-II (n=934)** | 496 (53%) |  |
| Age <70 | 334 (52%) | 1.00 (reference) |
| Age 70-74 | 91 (51%) | 0.98 (0.75-1.28) |
| Age ≥75 | 71 (61%) | 1.45 (1.07-1.98) |
| **III-IV (n=608)** | 508 (84%) |  |
| Age <70 | 339 (83%) | 1.00 (reference) |
| Age 70-74 | 102 (83%) | 1.19 (0.87-1.63) |
| Age ≥75 | 67 (89%) | 1.28 (0.88-1.87) |
| **Resection margin status** | 78 (85%) |  |
| **R0 (n=1335)** | 815 (61%) |  |
| Age <70 | 533 (60%) | 1.00 (reference) |
| Age 70-74 | 162 (60%) | 1.01 (0.81-1.25) |
| Age ≥75 | 120 (70%) | 1.45 (1.13-1.87) |
| **R1-R2 (n=207)** | 189 (91%) |  |
| Age <70 | 140 (91%) | 1.00 (reference) |
| Age 70-74 | 31 (94%) | 1.94 (0.95-3.96) |
| Age ≥75 | 18 (90%) | 0.91 (0.44-1.86) |

* Adjusted for sex, education, tumor histology, pathological tumor stage, and resection margin status.

Spline function of survival time with 4 knots.

**Table 7. Hazard ratios (HR) and 95% confidence interval (CI) of age in relation to mortality after esophagectomy for esophageal cancer.**

|  | **Unadjusted** | **Adjusted*** |
| --- | --- | --- |
|  | **HR (95% CI)** | **HR (95% CI)** |
| **90-day all-cause mortality** |  |  |
| **Age**^^^ |  |  |
| **<70** | 1.00 (reference) | 1.00 (reference) |
| **70-75** | 1.14 (0.79-1.65) | 1.11 (0.76-1.61) |
| **75+** | 2.08 (1.47-2.94) | 2.06 (1.45-2.92) |
|  |  |  |
| **5-year all-cause mortality** |  |  |
| **Age^§^** |  |  |
| **<70** | 1.00 (reference) | 1.00 (reference) |
| **70-74** | 1.01 (0.88-1.17) | 1.04 (0.90-1.20) |
| **≥75** | 1.36 (1.17-1.59) | 1.26 (1.07-1.47) |

* Adjusted for sex, education, Charlson comorbidity index, tumor histology, pathological tumor stage, and resection margin status.

^ Linear time.

§ Time with 4 knots.

a)b)

**Figure 1. Probability of 5-year disease-specific mortality after esophagectomy for esophageal cancer. a) Probability vs age at surgery for three time points after esophagectomy for esophageal cancer; b) Probability vs years from surgery for three age groups.**
